# Supplementary material for: A hydro-osmotic coarsening theory of biological cavity formation
Source: PLoS Comput Biol. 2021 Sep 3;17(9):e1009333. doi: 10.1371/journal.pcbi.1009333 (PMC8445475; doi:10.1371/journal.pcbi.1009333)
Supplement: S1 Table — (PDF) [file pcbi.1009333.s002.pdf]

S1 Table

Name	Symbol	Units [2D]	Range [3D]	Units [3D]
Thermal energy per unit mole	RT	$N.m.mol^{-1}$	2.4×10^3	$N.m.mol^{-1}$
Cell concentration [1] (K^+)	c_0	$mol.m^{-2}$	100	$mol.m^{-3}$
Hydrostatic pressure [2]	P	$N.m^{-1}$	$10 - 100$	$N.m^{-2}$
Osmotic pressure	π_0	$N.m^{-1}$	10^5	$N.m^{-2}$
Tension [3]	γ	N	5×10^{-4}	$N.m^{-1}$
Contact angle	θ	-	$\frac{\pi}{3}$	-
Contact tension	γ_c	N	$\sim \gamma$	$N.m^{-1}$
Intercellular space width [4]	e_0	nm	50	nm
Bridge length	ℓ	μm	$0.1 - 10$	μm
Typical lumen length	L_0	μm	$0.1 - 10$	μm
Total length	$\mathcal{L}_0$	μm	$10 - 100$	μm
Water viscosity	η	$N.s.m^{-1}$	10^{-3}	$N.s.m^{-2}$
Water permeability [5]	λ_v	$m^2.s^{-1}.N^{-1}$	7.32×10^{-13}	$m^3.s^{-1}.N^{-1}$
Solute permeability coefficient [6]	λ_s	$mol^2.N^{-1}.s^{-1}.m^{-2}$	10^{-8}	$mol^2.N^{-1}.s^{-1}.m^{-3}$
Solute diffusion constant (KCl) [7]	D	$m^2.s^{-1}$	2.10^{-9}	$m^2.s^{-1}$
Laplace/Osmotic pressures ratio	$\epsilon = \frac{\gamma \sin\theta}{L_0 \Pi_0}$	-	$10^{-2} - 10^{-3}$	–
Effective hydrodynamic friction	$\kappa_v = \frac{e_0^3}{12\eta}$	$m^4.N^{-1}.s^{-1}$	1.04×10^{-20}	$m^5.N^{-1}.s^{-1}$
Pressure screening length	ξ_v	μm	84	μm
Concentration screening length	ξ_s	μm	14	μm
Active pumping flux	j^a	$mol.s^{-1}.m^{-1}$	2.47×10^{-10}	$mol.s^{-1}.m^{-2}$
Solute time	τ_s	s	2	s
Water time	τ_v	s	7	s
Diffusion time	$\tau_D = \frac{\ell_0^2}{D}$	s	10^{-2}	s

Table 1: Symbols, values and units.

References

1. Powers RD, Tupper JT. Developmental changes in membrane transport and permeability in the early mouse embryo. Dev Biol. 1977;56(2):306–315. doi:10.1016/0012-1606(77)90272-X.

2. Maître JL, Niwayama R, Turlier H, Nédélec F, Hiiragi T. Pulsatile cell-autonomous contractility drives compaction in the mouse embryo. Nat Cell Biol. 2015;17(7):849–855. doi:10.1038/ncb3185.

3. et al JLM. Asymmetric division of contractile domains couples cell positioning and fate specification. Nature. 2016;536(7616):344–348. doi:10.1038/nature18958.

4. Fenz SF, Merkel R, Sengupta K. Diffusion and Intermembrane Distance: Case Study of Avidin and E-Cadherin Mediated Adhesion. Langmuir. 2009;25(17):1074–1085. doi:10.1021/la803227s.

5. et al KE. Channel-Dependent Permeation of Water and Glycerol in Mouse Morulae. Biol Reprod. 2006;74(4):625–632. doi:10.1095/biolreprod.105.045823.

6. Dasgupta S, Gupta K, Zhang Y, Viasnoff V, Prost J. Physics of lumen growth. Proc Natl Acad Sci USA. 2018;115(21):E4751–E4757. doi:10.1073/pnas.1722154115.

7. Friedman AM, Kennedy JW. The Self-diffusion Coefficients of Potassium, Cesium, Iodide and Chloride Ions in Aqueous Solutions. J Am Chem. 1955;77(17):4499–4501. doi:10.1021/ja01622a016.
