# Supplementary material for: A hydro-osmotic coarsening theory of biological cavity formation
Source: PLoS Comput Biol. 2021 Sep 3;17(9):e1009333. doi: 10.1371/journal.pcbi.1009333 (PMC8445475; doi:10.1371/journal.pcbi.1009333)
Supplement: Attachment — Submitted filename: RefReportTurlier.pdf [file pcbi.1009333.s011.pdf]

Report for the article « A hydro-osmotic coarsening theory of biological cavity formation » submitted to PLOS Computational Biology by Mathieu La Verge-Serandour and Hervé Turlier.

The article discusses an original model for the formation of lumens from a theoretical and numerical point of view and from a physical perspective. Especially, the authors investigate the role of ions exchange and water exchange between lumens in their growth dynamics, as well as the role of active pumping. The theory is based on a set of coupled differential equation describing the mass balances of each species within the cavities and the local balances along the channel connecting them. Two situations are at the center of the paper: the dynamics of two cavities and subsequent coalescence/coarsening/collapse, and the case of a string of cavities that coarsen into a single one. The dynamics observed are essentially investigated by changing the time scales and screening length scales of the dynamics which control the magnitude of the flows.

While I could not verify the lengthy mathematical discussion developed in the appendix and used in the first part of the manuscript, the results, arguments and methods provided in the article and in the appendix are convincing and give an extensive description of the dynamics which certainly will drive further experimental investigations for comparison. Consequently, I think the article should be considered for publication. Yet, some modifications should be applied to the text, especially about technical issues with the manuscript and additional discussion for the reader, as listed below.

1) I have a hard time to relate the results on the "2-body" part of the article in section "Coupled dynamics for two lumens" to the results shown in the part dedicated to the evolution of the 1D chain of lumens. Could the authors try to relate more the two sections of their manuscript? Is there any insight gained from the 2-body problem which can directly be applied to the N-body problem discussed in the chain section?

2) I guess that a technical issue with the creation of the final PDF has occurred during the submission process. Especially, I noted:
   - Issues with the references to the figures in the main text (as far as I could guess, with Figs 4,5 and 6)
   - Issues with the labelling of sub-figures which does not corresponds to the text in the article.

   Because of that, it is hard to read through the second part of the manuscript, to comment and criticize of the article itself, and I could only guess the correct figures. I suggest checking all references to the figures according to the two points here above.

3) I would move the definition of the coefficient μ introduced in the Appendix and in page 4/20 of the manuscript where it is used.

4) I could not find the definition of $\delta\bar{P}$ and of $\delta\bar{C}$ in the main text. Also, how would it differ from the definition of ΔP and ΔC?

5) I might have missed the information but what is the value of the active pumping j_a in Fig2? Indeed, it is difficult to place it in the context of Fig3b. Similarly, what are the initial size L_1 and L_2 in the case of Fig2?

6) On a more general aspect, I can see from the theory that the cavities have their position fixed but cannot move. Would it be realistic to expect such a motion and if yes, would it significantly alter the overall picture drawn by the theory? Can the authors comment on that point?
