# Supplementary material for: A hydro-osmotic coarsening theory of biological cavity formation
Source: PLoS Comput Biol. 2021 Sep 3;17(9):e1009333. doi: 10.1371/journal.pcbi.1009333 (PMC8445475; doi:10.1371/journal.pcbi.1009333)
Supplement: Attachment — Submitted filename: PLOS_answers.pdf [file pcbi.1009333.s012.pdf]

We thank the reviewers for their careful assessment of our manuscript and constructive remarks. We answer below their questions and tried to address all their suggestions in the novel version of the manuscript.

Reviewer 1

This is a comprehensive theoretical and computational study of the collective dynamics of lumens, as driven by flow of water and osmolytes between cavities and by pumping of osmolytes. The authors predict whether coarsening in a single lumen occurs and discuss applicability of their results to experiments. The work and results are of potential high interest to developmental biologists, to biophysicists, and to applied mathematicians. It is definitely worth publishing, though the clarity of the writing needs to be improved.

We thank the reviewer for his positive assessment of our work and careful review. We modified the manuscript to clarify all the points raised.

CONTENTS

- *What would change in a more realistic 3D geometry, with lumens of finite size and bridges in the form of tubes?*
  Answer: We expect qualitatively analog results in a more realistic 3D geometry, where lumen take the shape of two symmetric spherical caps apposed against each other, and the intercellular space is a 2D sheet (not a tube) (see *Appendix, Sec. 1.2 Bridge equations*). In particular, the minor influence of passive osmotic effects found on the dynamics, the transition toward a coalescence-dominated regime for large enough active pumping, and the influence of pumping patterning on the final lumen position are generic results that shall not to depend on the dimension. For 3D geometry, in the hydraulic coarsening regime, we expect a scaling law $N \sim t^{-3/4}$ for 3D-lumens living on a 2D surface Ref [53,58, 59]. We predict a scaling law $N \sim t^{-1}$ for the coalescence-dominated regime, independently of the dimension (eq. 50 in Appendix). Performing simulations in 2D allowed us to explore extensively the parameter space, which would have been technically and computationally very challenging in 3D.

- *L186. Why a factor of 2 before the sum of L_i?*
  Answer: Lumens are parametrized by their half-length L_i (as depicted on Fig. 1D) while bridges are parametrized by their (total) length \ell$_{ij}$. We have now made the half-length definition explicit in the Fig.1 caption and in the text (line 88).

- *L225. What is the meaning of the timescale of 1s to which tau_s is compared? Normally one should compare tau_s to another timescale defined by problem parameters.*
  Answer: We thank the reviewer for pointing this out. τ_s was indeed meant to be compared with τ_v, and we changed the text accordingly (Line 264). We checked this condition explicitly by generating below simulations with the same parameters as in Fig. 4D but with $\tau_v = 10s$ instead of 1s. We show on **Response Fig. 1** that curves with the same ratio τ_s/τ_v overlap:

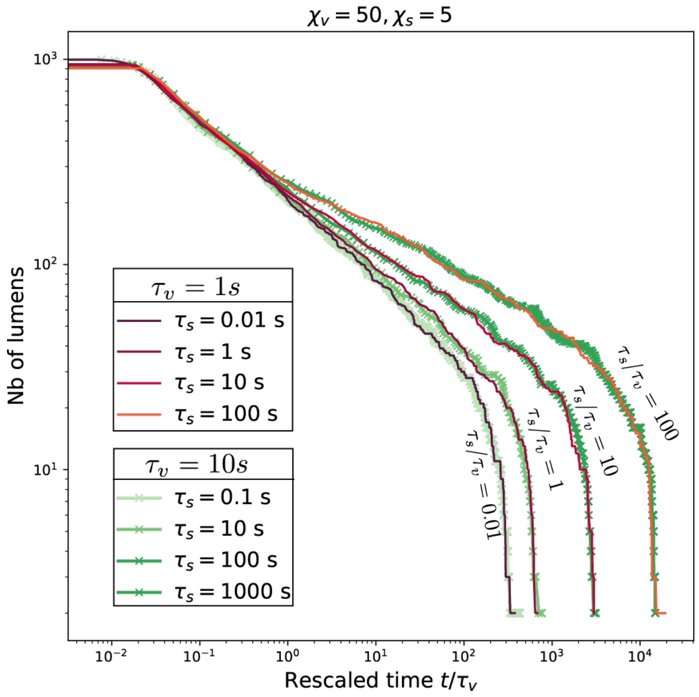

Response Fig. 1: Plot of the number of lumens versus rescaled time t/τ_v, with two sets of relaxation times (τ_s, τ_v). Parameters for all simulations are $\chi_v = 50$, $\chi_s = 5$, N(0)=1000, $j^a = 0$. Curves in shades of red corresponds to panel 4D of the main manuscript, with $\tau_v = 1s$. Curves in shades of green have $\tau_v = 10s$, such that the ratio τ_s/τ_v is the same than a red-shade curve. Each curve is one simulation.

- *How relevant is this work to other types of lumens?*
  Answer: We believe that our model is generic to describe the collective dynamics of interconnected cavities pressurized by a surface tension and whose volume is controlled osmotically. The model was not originally designed to describe the interaction between apical lumens, as they are supposedly isolated from each other by tight junctions (Line 12). However, we became aware of systems (such as pancreatic organoids, A. Bausch's personal communication), where apical lumens might interact by fluid exchange in a similar manner. We are confident that more and more biological cavities interacting in an analog fashion will be identified experimentally in the next years. Our model may also be relevant to describe the collective dynamics of cells that directly exchange fluid through canals such as germ cells during Drosophila oogenesis (*Alsous et al. PNAS 2021* Ref 39) or in *C elegans* (Chartier et al. *Nat Phys 2021* Ref 40). We note that no osmotic effect was considered in those manuscripts, while the volume of cells is known to be first and foremost regulated osmotically. Our model justifies why a purely hydraulic approach might be sufficient, in first approximation, to describe such phenomena, which does not seem to us obvious a priori.

WRITING

- *The beginnings of the abstract and introduction are too technical. Please define (or refrain from using): apical, basolateral, blastocoel…*
  Answer: We have changed the abstract and introduction to better define such terms, which are yet ordinary in biology.
- *The author summary should be written for a scientist that is far from the field.*
  Answer: We have modified the author summary to make it more accessible to scientists far from the field.

- *In caption of Fig. 1: A_i and mu may be omitted, otherwise they need to be defined*
  Answer: We have corrected by removing $L_i = \sqrt{\mu A_i}$ from the caption.
- *L78. Why isn't sin(theta) absorbed in mu? What is the definition of mu?*
  Answer: $\mu = \sin^2\theta\,/(2\theta - \sin 2\theta)$ and $\nu = \theta/\sin\theta$ are functions of the contact angle theta that appeared recurrently due to the semicircular shape of lumens. We defined them by convenience in the Appendix. We now added their definition in the main text (Lines 79 and 90).
- *L87. Perhaps the definition of L_i should appear earlier.*
  Answer: We have introduced the definition of the lumen half-length L_i before Eq (1) (Line 88)
- *P4. Briefly explain why bridge thickness is considered to be constant.*
  Answer: This is a simplifying hypothesis, where we assume that cadherin molecules are stiff and concentrated enough to maintain the two apposed membranes at a fixed distance e_0. This justification appears explicitly in the Appendix (see *Appendix, Sec. 1.2 Bridge equations)*. Experimentally in mouse embryos (Ref 18) no measurable variation of the intercellular distance can be detected.
- *P5. It is not well thought to call dimensionless numbers 'screening lengths'. Why not 'screening numbers'?*
  Answer: It is true that the ratio χ_v and χ_s correspond to numbers, as they give the typical average number of lumens, that a given lumen can exchange with. That is an excellent suggestion that we have now implemented in the manuscript, denoting them screening numbers.
- *Eqs. 6-8, why mix dimensionless quantities and time? This may be misleading.*
  Answer: This was a choice to emphasize the fact that τ_v was associated with lumen size relaxation, and τ_s with solute relaxation. We have now written Eqs. 6-8 such that the time is non-dimensionalized by the typical solute time τ_v.
- *In insets of Fig.3A, the differences in size between lumens 1 and 2 are too small to be visible.*
  Answer: We have corrected their sizes to make them more visible, and inverted panels A and
- *P7. A brief description of coalescence events is needed.*
  Answer: We added a description of coalescence events, found in Lines 198-204:
  " *Above some pumping rate a fourth dynamical 198 regime appears, where both lumens grow faster than they exchange their content, 199 leading to a fast decrease of the bridge length and lumen collision. Upon collision, two 200 lumens are merged: this is referred as coalescence, and may be observed in late stages of 201 mouse blastocoel embryo formation [18] but also in droplet coarsening [48,49]. As we do 202 not model the profile of the interface as in [50] but rather consider a coarse-grained 203 description of the lumens, we consider the coalescence events to be instantaneous, such 204 that the total mass of colliding lumens is conserved."*
- *L247. What does \bar{L}_{ij} stand for?*
  Answer: this length was denoting the distance between the centers of mass of the two lumens i, j, $L_{ij} = L_i + L_j + l_{ij}$, which is constant as the lumens are assumed not to move. We changed the text to make this point clearer (Line 288). Note that upper bars stand for nondimensionalization.

Reviewer 2

I read this paper by Le Verge–Serandour and Turlier with interest. The problem of lumen formation is an interesting biophysical problem, with numerous unknown subtopics that require some detailed understanding. The treatment by the author is quite elegant mathematically, and provides useful simplifications for the physical problem. The paper is nicely done and generally clear, and can be published. However, I find some simplifications in the current form are unrealistic biologically. Since this is a journal about computational biology, the treatment can include more biological detail. In particular models of active ion flux exist. The paper can improve substantially with some revision and additions.

We thank the reviewer for her/his comments on our manuscript and overall positive assessment. We disagree, on the “unrealistic” (or uncommon) character of our physical model assumptions in regard to the biologically systems studied. We believe there might be a misunderstanding of the model details and therefore added more explanations in the text of model hypotheses, limitations and possible extensions.

Contents

A major problem is, j^a or the ion flux is not a constant. j^a should depend on concentration differences, and energy input by cells (it's a pump), and potentially tension in the membrane. There are models of j^a already in the literature, for example, Jiang & Sun, Biophysical J. 2013, and more detailed treatment in subsequent papers with different ionic species.

Answer:

We think there might be an misunderstanding in the notation we used. Our assumption is to decompose the ion flux per unit length into two contributions (Appendix, top of page 3): the first is a passive flux, that we will denote j^p, and is proportional to the chemical potential difference across the membrane $\Delta\mu = RTlog(\frac{c_0}{c})$, which depends explicitly on concentrations, and the second is an active flux, j^a, which corresponds to a pumping rate in mol/s per unit length. This active flux depends on energy input by the cell indeed, although not explicitly modeled, and we supposed it constant across the network. This assumption of constant active pumping rate is very classical, and is found in most theoretical models of volume control based on the well-established pump-leak mechanism (*Tosteson & Hoffmann 1960* Ref 61): *Hoppensteadt 2002* (Ref 62), *Armstrong 2003* (Ref 63), *Mori 2012* (Ref 64), *Kay 2017* (Ref 65). Importantly, this assumption allows these authors, and us, to study the behavior of the system as function of this control parameter, which is directly tuned by the cell metabolism, as mentioned by the reviewer. Although various additional feedbacks on the active and passive fluxes might be added, we believe they are generally specific to a biological system and should therefore be justified by clear experimental evidence. Following the results that may be found in the Appendix 2 of Ref 65 (*Kay 2017*), it was shown that a more complex active pumping flux, that explicitly depends nonlinearly on the concentrations of Na^+ and K^+, does not change the shape of concentration curves (or membrane potential or volume) as function of the pumping rate: volume control does therefore not critically depends on such feedback. This is a strong justification that a more complicated j^a would not change qualitatively the results of our model, that intends to remain as generic as possible, and which predicts already a rich behavior and universal scaling laws, although based on simplifying hypotheses. Based on our observations in mouse embryos (*Dumortier et al. 2019* Ref 18) other feedbacks do not appear to be needed. We however cite the mentioned paper *Jiang & Sun Biophysical J. 2013* (Ref 60) in the discussion of our model (Line 377) to indicate that feedbacks might be added. More generally, we made a more fundamental simplifying hypothesis in our model, which is actually exactly analog to *Jiang & Sun Biophysical J. 2013*: we consider a single “ion”, neglect charges, electro-chemical potentials and the membrane potential. We fully acknowledge such approximation from the beginning of our manuscript (Lines 63-65) and discuss its limitations lengthily at the end (Lines 376-393).

The active water flux across an epithelial barrier has been measured, e.g., see Li et al, Journal of Cell Science, 2020. For constant lumen osmolarity, the water flux was found to be maximum at Delta P=0, and decreases with increasing Delta P. The reason for this is can be traced to the properties of j^a. It is true that a detailed theory for j^a is lacking, but there has been work on this. The expression of j^a as a function of ion concentration will change the results qualitatively. Can the authors add some results with more realistic j^a?

Answer: The water flux in our model is explicitly proportional to ΔΠ-ΔP (see Eq. 3 for a bridge in the main text and Eq. 2 in the Appendix for a lumen), and therefore is also maximum for ΔP=0. We believe again

that there may be a misunderstanding in the derivation of our model, which is generic and in line with previous theoretical studies in the field, such that its details were therefore deferred to the Appendix. We thank the reviewer for mentioning to us the review *Li et al. Journal of Cell Science 2020*, that we added to the bibliography (Ref 10). We note however the water fluxes we consider here are through a single cell membrane as we did not model explicitly cellular compartments. The cellular compartment is simplified as a barostat and chemostat (Line 84). As such, measurements of water fluxes across an epithelium are difficult to compare directly with our model.

In the presented physics, the rate at which l decreases is solely due to lumen expansion (Eq. 8) from water inflow. But another importance piece seems to be the break down of tight junctions that keep the junction zipped. One can imagine a scenario where P builds up sufficiently so the tension increases, and therefore unzips the tight junction. The rate of unzipping may depend on various factors. Where does this physics come in?

Answer: We explain explicitly in the introduction of our manuscript that our theory concerns primarily lumens that form at the basolateral side of cell aggregates. Tight junctions are exclusively located at the apical side of polarized cells, and therefore don't need to be broken for the coarsening process here, as they are absent from junctions between adhesive bridges and lumens (in a 1D chain, tight junctions would be seals located at the two ends of the chain, and we now precise it on Lines 222-223). Tight junctions are represented by red symbols on Fig.1A, and we now explicitly added their mention in the corresponding legend. The unzipping of adhesive contacts (cadherins) is already taken into account by tension balance at lumen-bridge junctions, that defines the contact angle θ : it does enter dynamic equations (through μ and ν parameters), but is assumed constant (see answer below) so it is decoupled from lumen expansion.

In a biologically realistic lumen, is it true that gamma, gamma_c and therefore mu are constants spatially and temporally within the lumen? Does the tension change with time? I would think there is an active adjustment process. Is the assumption with constant theta realistic?

Answer: In our model, we assume all tensions, and hence all contact angles, constant in time and homogeneous in space. The effect of inhomogeneity in space of cortical tensions was already studied in details in a former manuscript: *Dumortier et al. Science 2019* (Ref 18). Here, we assume the coarsening process very slow compared to cortex mechanics relaxation (in mouse embryos the coarsening process takes about 10 hours, to compare with minutes timescales cortical relaxation), such that potential viscous contributions to cortical tensions will be largely negligible. We believe therefore that our assumption of constant tensions is well justified and now discuss in more details this hypothesis (Lines 363-375). We note that considering explicitly the viscous contribution to cortical tension generated by rapid lumen deformations was already done in *Dasgupta et al. 2018* (Ref 35) for a single (experimentally apical but modeled as basolateral) lumen, and may lead to lumen shape oscillations on short timescales. The study of potential short timescale oscillations in a collective lumen context is undoubtedly interesting but beyond the scope of this manuscript, which brings already a dense amount of novel results for the coupled dynamics on longer timescales.

What happens when 2 lumens collide and l for the bridge goes to zero? It seems that some assumptions break down here. At this moment, L_1+L_2=1, and you still have 2 lumen, how does this transition to 1 lumen? I didn't find details about this. Right now there would be a jump in overall mass of the lumen (Fig. 3A). This jump doesn't happen in reality, and the new lumen is filled slowly. How does this change the result for N(t) in Fig. 4. Does this take some time, and therefore change the time scale?

Answer: We described the details of lumen collision during simulation in the Appendix, Part 3 *Chain integration*. Specifically, when the bridge length is smaller than a given threshold l_{merge} (Appendix Eq. 63), the two adjacent lumens merge into a new lumen such that the total area is conserved (Appendix Eq. 65), and its position is the area-weighted average of the two former lumens positions (Appendix Eq. 64).

Such instantaneous processes indeed generate jumps, that may be detected in Movies S2, S3. Experimentally in mouse embryos, coalescence processes, when detected, are much quicker than the coarsening (see *Dumortier et al. 2019* Ref 18), which justifies our approximate treatment. Note that in Fig.4 BCD, there is no active pumping, and therefore no coalescence observed. Scaling laws in physics are always obtained as statistical averages of a large number of repetitions of the same dynamic process: in Fig. 4, and in the subsequent Fig. 5 with active pumping (and coalescence) plotted curves are averages of a minimum of 20 simulations with 1000 lumens, as stated in the legend, and therefore single jumps are smoothed.

Reviewer 3

The article discusses an original model for the formation of lumens from a theoretical and numerical point of view and from a physical perspective. Especially, the authors investigate the role of ions exchange and water exchange between lumens in their growth dynamics, as well as the role of active pumping. The theory is based on a set of coupled differential equation describing the mass balances of each species within the cavities and the local balances along the channel connecting them. Two situations are at the center of the paper: the dynamics of two cavities and subsequent coalescence/coarsening/collapse, and the case of a string of cavities that coarsen into a single one. The dynamics observed are essentially investigated by changing the time scales and screening length scales of the dynamics which control the magnitude of the flows. While I could not verify the lengthy mathematical discussion developed in the appendix and used in the first part of the manuscript, the results, arguments and methods provided in the article and in the appendix are convincing and give an extensive description of the dynamics which certainly will drive further experimental investigations for comparison. Consequently, I think the article should be considered for publication. Yet, some modifications should be applied to the text, especially about technical issues with the manuscript and additional discussion for the reader, as listed below.

We thank the reviewer for the positive assessment of our manuscript and highly relevant remarks. We tried to provide convincing answers to all questions below.

Contents

1. *I have a hard time to relate the results on the "2-body" part of the article in section "Coupled dynamics for two lumens" to the results shown in the part dedicated to the evolution of the 1D chain of lumens. Could the authors try to relate more the two sections of their manuscript? Is there any insight gained from the 2-body problem which can directly be applied to the Nbody problem discussed in the chain section?*

   Answer:

   We thank the reviewer for pointing out the lack of transition between the two parts of our manuscript. We now added a short paragraph to summarize the results learned from the 2-body system (Lines 208-216), and draw more explicit comparisons while discussing the N-body system. In particular, the 2-lumen model allows us:

   - To study explicitly the competition between hydraulic and osmotic fluxes in setting the instantaneous direction of the net water flux, predicting already a minor role for passive osmotic heterogeneities in the coarsening, that we confirmed on N-lumen systems with N>>1.
   - To study the effect of active pumping, which can prevent the system from a collapse driven by passive water permeation, and if sufficiently large, may drive the coalescence of two lumens. We find similar average behaviors on the N-body system.
   - To define a typical hydraulic exchange time, T_h (section 2.1.1 of Appendix 1) and a typical pumping time, T_p (section 2.1.2 of Appendix 1). From a single lumen, or two isolated lumens, one can show these times.

2. *I guess that a technical issue with the creation of the final PDF has occurred during the submission process. Especially, I noted:*

a. *Issues with the references to the figures in the main text (as far as I could guess, with Figs 4,5 and 6)*
   Answer: It was indeed an issue of labelling in the Latex file, that is now corrected.
b. *Issues with the labelling of sub-figures which does not corresponds to the text in the article.*
   Because of that, it is hard to read through the second part of the manuscript, to comment and criticize of the article itself, and I could only guess the correct figures. I suggest checking all references to the figures according to the two points here above.
   Answer: We apologize for this unexpected issue during compilation of our Latex file.

3. *I would move the definition of the coefficient μ introduced in the Appendix and in page 4/20 of the manuscript where it is used.*
   Answer: We have accordingly recalled the definitions of the geometrical coefficients μ and ν where they first appear in the manuscript.
4. *I could not find the definition of $\delta\bar{P}$ and of $\delta\bar{C}$ in the main text. Also, how would it differ from the definition of ΔP and ΔC?*
   Answer: The capital deltas refer to pressure and concentration differences between two lumens and are defined in the main text (Lines 151 and 153). Smaller deltas correspond to pressure and concentration differences with respect to the external medium: $\delta P_i = P_i - p_0$ and $\delta C_i = C_i - c_0$ defined on lines 78 and 89 respectively for the lumens, and on line 102 for bridges: $\delta p(x) = p(x) - p_0$ and $\delta c(x) = c(x) - c_0$. We now explicitly mentioned the capital (resp. lowercase) notation for lumens (resp. bridges) (Lines 74-75).
5. *I might have missed the information but what is the value of the active pumping ja in Fig2? Indeed, it is difficult to place it in the context of Fig3b. Similarly, what are the initial size L1 and L2 in the case of Fig2?*
   Answer:
   a. We did not specify the value of $j^a = 0$ in Fig. 2 as we intended to study the competition between two passive effects on controlling the net flux direction (concentration vs pressure difference). However, we understand that it could be misleading, thus we added this information in the caption of Fig. 2.
   b. In Fig. 3, the lumens start at osmotic equilibrium, thus this corresponds to $\Delta_C = 0$ in Fig. 2. We now added this in the caption of Fig. 3. Note that we inverted the order of panels A and B in Fig.3 for the sake of readability.
   c. In Fig. 2, the initial sizes of lumens are not fixed, but rather directly their pressures P_1, P_2, as this is more general to describe hydraulic fluxes (lumens could have the same size but different tensions for instance). In panel C, the initial lengths were L1=0.909, L2=0.812, and are now modified to correspond to Fig.3 initial conditions (L1=1, L2=1.1).

   On a more general aspect, I can see from the theory that the cavities have their position fixed but cannot move. Would it be realistic to expect such a motion and if yes, would it significantly alter the overall picture drawn by the theory? Can the authors comment on that point?
   Answer: This is a very good question. In the framework of thin films, droplet coarsening shall indeed include, in all genericity, a translational motion of droplets that is generated by asymmetric fluxes $\frac{dX}{dt} = C_x.(J_R - J_L)$ where C_x is a mobility coefficient, and J_L, J_R are the outgoing left and right fluxes (see Refs 50-52). Here, we hypothesized a fixed center of mass for the lumens, first motivated by experimental observations in mouse embryos, where no motion of microlumens could be observed (Ref 18). While we tried to derive a similar mobility coefficient, our theory, our assumption of symmetric lumen shapes prevents symmetry breaking that would allow for translational motion, even adding an *ad hoc* adhesive friction to oppose translational motion. We do not believe that this simplification affect our results, as it was shown in thin films with numerical simulations and theoretical predictions that the scaling law exponent $N(t) \sim t^{-2/5}$ remains unaffected by the motility of droplets [see in Ref 50, Eq. 4.22 page 100 and Fig. 17]. Corrections due to motility appear only in the prefactor of this scaling law, a prefactor that we do not pretend to predict here. We now added an explicit explanation for this hypothesis in the main text (Lines 134 to 141).
