# Supplementary material for: A hydro-osmotic coarsening theory of biological cavity formation
Source: PLoS Comput Biol. 2021 Sep 3;17(9):e1009333. doi: 10.1371/journal.pcbi.1009333 (PMC8445475; doi:10.1371/journal.pcbi.1009333)
Supplement: Attachment — Submitted filename: RefReportTurlier2.pdf [file pcbi.1009333.s013.pdf]

Second referee report for the article « A hydro-osmotic coarsening theory of biological cavity formation » by Mathieu Le Verge-Serandour and Hervé Turlier.

I want to thank the authors for diligently addressing the comments of my first report. This second version of the article answers all the questions and criticisms I had previously, especially regarding the possible motion of the lumens and the relation between the two main sections of the manuscript, and also fixes all the technical issues I raised previously.

As stated in my previous report, this article will attract the attention it deserves and consequently, the manuscript should be published. I invite the authors to consider the following minor remarks to increase even more the overall quality of their manuscript.

- Is the reference to “S2 FigC” at line 174 is correct? Indeed, Figure S2 does not contain a subfigure C and does not relate to the discussion in that part of the manuscript.
- Additionally, I couldn’t find a correct reference to figure S2 anywhere in the text. The same comment applies for Figure S3A. If I simply missed the references, I invite to authors to disregard this comment.
- Is overlap allowed when placing the lumens along the chain (see line 223/224). If yes, I invite the authors to state it in this part of the manuscript as it would change the effective distributions of lumen area.
- 3A should read Fig3A at line 243.
- I assume the authors refer to the pressure screening length at line 248. If yes, the authors should correct the sentence.
- I invite the authors to provide a longer description for the SI figures. Indeed, it was challenging to understand the claims of the authors in some parts of the manuscript. For instance, see line 269 when referring to figure S3C.
- Finally, in Fig6C, it should be made clear that the red dashed curve corresponds to the unperturbed case in Fig6B in contrast to all other curves. I invite the authors to explain this explicitly in the figure caption.
